# Childhood Pompe disease: clinical spectrum and genotype in 31 patients

**DOI:** 10.1186/s13023-016-0442-y

**Published:** 2016-05-18

**Authors:** C. I. van Capelle, J. C. van der Meijden, J. M. P. van den Hout, J. Jaeken, M. Baethmann, T. Voit, M. A. Kroos, T. G. J. Derks, M. E. Rubio-Gozalbo, M. A. Willemsen, R. H. Lachmann, E. Mengel, H. Michelakakis, J. C. de Jongste, A. J. J. Reuser, A. T. van der Ploeg

**Affiliations:** Pompe Center and Center for Lysosomal and Metabolic Diseases, Erasmus MC University Medical Center, Room Sb-1629, P.O. BOX 2060, 3000 CB Rotterdam, The Netherlands; Centre for Metabolic Disease, University Hospital Gasthuisberg, KU Leuven, Leuven, Belgium; Department of Pediatrics, Hospital “Dritter Orden”, Munich, Germany; NIHR Biomedical Research Centre, UCL Institute of Child Health and Great Ormond Street Hospital, London, UK; Division of Metabolic Diseases, Beatrix Children’s Hospital, University of Groningen, University Medical Center Groningen, Groningen, The Netherlands; Department of Pediatrics and Laboratory Genetic Metabolic Diseases, Maastricht University Medical Center, Maastricht, The Netherlands; Department of Pediatric Neurology, Radboud University Medical Center, Nijmegen, The Netherlands; Charles Dent Metabolic Unit at University College London Hospitals, London, UK; Villa Metabolica, Centre for Pediatric and Adolescent Medicine, Mainz, Germany; Department of Enzymology and Cellular Function, Institute of Child Health, Aghia Sophia Children’s Hospital, Athens, Greece; Department of Pediatrics, Division of Pediatric Respiratory Medicine, Erasmus MC University Medical Center, Rotterdam, The Netherlands

**Keywords:** Pompe disease, Childhood, Clinical spectrum, Genotype, Natural course

## Abstract

**Background:**

As little information is available on children with non-classic presentations of Pompe disease, we wished to gain knowledge of specific clinical characteristics and genotypes. We included all patients younger than 18 years, who had been evaluated at the Pompe Center in Rotterdam, the Netherlands, between 1975 and 2012, excluding those with the classic-infantile form. None were treated with enzyme replacement therapy at the time of evaluation. We collected information on first symptoms, diagnosis, use of a wheelchair and/or respirator, and enzyme and mutation analysis and assessed muscle strength, pulmonary function, and cardiac parameters.

**Results:**

Thirty-one patients participated. Median age at symptom onset was 2.6 years (range 0.5–13y) and at diagnosis 4.0 years. Most first problems were delayed motor development and problems related to limb-girdle weakness. Fatigue, persistent diarrhea and problems in raising the head in supine position were other first complaints. Ten patients were asymptomatic at time of diagnosis. Five of them developed symptoms before inclusion in this study. Over 50 % of all patients had low or absent reflexes, a myopathic face, and scoliosis; 29 % were underweight. Muscle strength of the neck flexors, hip extensors, hip flexors, and shoulder abductors were most frequently reduced. Pulmonary function was decreased in over 48 % of the patients; 2 patients had cardiac hypertrophy. Patients with mutations other than the c.-32–13T > G were overall more severely affected, while 18 out of the 21 patients (86 %) with the c.-32–13T > G/‘null’ genotype were male.

**Conclusions:**

Our study shows that Pompe disease can present with severe mobility and respiratory problems during childhood. Pompe disease should be considered in the differential diagnosis of children with less familiar signs such as disproportional weakness of the neck flexors, unexplained fatigue, persistent diarrhea and unexplained high CK/ASAT/ALAT. Disease presentation appears to be different from adult patients. The majority of affected children with *GAA* genotype c.-32–13T > G/‘null’ appeared to be male.

## Background

Pompe disease, also known as acid maltase deficiency or glycogen storage disease type 2 (OMIM 232300), is a lysosomal storage disorder that presents as a progressive myopathy in which deficiency of the enzyme acid α-glucosidase (EC 3.2.1.20) causes glycogen to accumulate in lysosomes. Ultimately, this leads to cell destruction [1–3]. In 1932, J.C. Pompe first described the classic-infantile form of the disease [4]. Classic infantile patients characteristically present shortly after birth with generalized and severe muscle weakness and with hypertrophic cardiomyopathy. They do not reach major milestones like walking and usually die within their first year of life [5, 6]. Later, other forms were reported and Pompe disease appeared to be a continuous spectrum of closely related phenotypes with the classic-infantile form at the most severe end of the spectrum. In the literature, milder phenotypes are referred to as childhood, juvenile, adult and late-onset. Patients with these non-classic variants of Pompe disease usually have no hypertrophic cardiomyopathy and present with a more slowly progressive limb-girdle muscle weakness, which eventually results in wheelchair dependency, respirator need, and shortened life expectancy [3, 7–17].

While many publications have described the natural history of the disease in classic-infantile and adult Pompe patients [5, 6, 12, 14–16], there is little information on presenting signs and symptoms in children who do not fulfill the criteria of classic-infantile Pompe disease [18].

To gain knowledge of the presentation of Pompe disease in children and to describe their specific clinical characteristics, we set up an observational study to collect information on disease symptoms, the distribution and severity of muscle weakness, physical limitations, lung function, cardiac structure and function, and genotypes of 31 children diagnosed with a non-classical presentation of Pompe disease.

## Methods

### Subjects

This observational study included all patients under the age of 18 years who had been diagnosed at or referred to the Pompe Center at Erasmus MC University Medical Center between 1975 and 2012. These patients came from the Netherlands and abroad, and had been diagnosed by measurement of the acid α-glucosidase activity in cultured fibroblasts, leukocytes or muscle biopsy specimens, and by mutation analysis. Patients with classic-infantile Pompe disease were not included in this study.

Thirty-one patients participated in this cross-sectional study. They were evaluated as part of studies approved by the Institutional Review Board (*n* = 28) or as part of routine clinical evaluation (*n* = 3). Medical history was obtained at first visit. Per patient, the following data were collected: gender, current age, geographic origin, first symptoms, age at first symptoms, age at diagnosis, wheelchair use, respiratory support, specific clinical findings (e.g. facial muscle weakness, bulbar muscle weakness, scoliosis, contractures, or muscle atrophy); functional impairments, spirometry and weight and height. Low body weight was defined as weight corrected for height if under 2SD for peers. Blood tests included measurement of creatine kinase (CK), aspartate aminotransferase (AST), alanine aminotransferase (ALT), and lactate dehydrogenase (LDH). A pediatrician and child neurologist performed clinical and neurological examinations in all patients. Only natural course data were included. None of the patients had been treated with enzyme replacement therapy.

### Muscle-strength testing

Muscle strength was assessed by hand-held dynamometry (HHD) (*n* = 24) and manual muscle-strength testing (MMT) (*n* = 24) [19, 20]. The following muscle groups were tested with HHD: neck flexors, shoulder abductors, elbow flexors, wrist extensors, hip flexors, hip abductors, knee extensors, knee flexors, and foot dorsal flexors. HHD scores (Newton) were expressed as percentages of the reference values (50^th^ percentile) for healthy peers [20]. All percentages were cumulated and divided by 9 to obtain a total HHD sum score expressed in percentage of normal.

MMT was performed according to the Medical Research Council guidelines [21] for the following muscle groups: neck flexors, neck extensors, deltoid muscles, biceps, triceps, wrist extensors, hip flexors, hip extensors, hip abductors, hip adductors, knee flexors, knee extensors, and foot dorsal and plantar flexors.

### Lung function testing

Lung function testing was performed by spirometry with the patients in upright-seated position (*n* = 28), and supine position (*n* = 23) according to ATS/ERS standards [22]. The highest outcome of three reproducible tests was used for analysis. The results were expressed as percentage of predicted and as a z-score, due to the paradigm shift towards z-scores, based on reference values corrected for age, length, gender and race [23]. A percentage lower than 80 % of predicted and a z-score below −1.64 were considered abnormal. Two patients were too young for reproducible spirometry to be reliable. Because of poor lung function six other patients were unable to perform testing in a supine position and one of them in either sitting or supine position.

### Cardiac assessment

In all patients, conventional Doppler, and 2D M-mode tracings were performed by an experienced sonographer (JP) according to the recommendations of the American Society of Echocardiography (Sonos 5500 ultrasound system, Philips, Best, the Netherlands). Standard 12-lead electrocardiograms were also made and analyzed by a pediatric cardiologist.

### Enzymatic and molecular assays

Acid α-glucosidase activity was measured in leukocytes [24] and in cultured skin fibroblasts [13] according to standard procedures, and was expressed in nmol/h/mg protein. The protein concentrations of cell homogenates was measured as described previously [25].

Genomic DNA was isolated from blood or cultured fibroblasts, and mutation analysis of the *GAA* gene was performed according to standard procedures [13, 26]. The severity of the mutations were rated using the format of Kroos et al. [27], and were examined for their effect on enzyme activity, quantity, and quality, in transfected cells after site directed mutagenesis [26]. In case of splice site mutations, the effect of the mutation was examined using real-time PCR in mRNA, isolated from the patients’ fibroblasts [27].

### Statistics

Demographic and clinical data were summarized using descriptive statistics including mean, SD, median, ranges, and percentages. As the data were not normally distributed, differences between groups were analyzed using a Mann–Whitney test. *P*-values < 0.05 were considered statistically significant. All statistical analyses were performed using SPSS for Windows version 21.

## Results

### Symptom onset and diagnosis

Thirty-one children participated in this cross-sectional study. Table 1 shows the patient characteristics and genotype. Twenty-two patients were male, nine were female. There were 17 patients from the Netherlands, 4 from Belgium, 5 from Germany, 3 from Greece, 1 from Great Britain, and 1 from the United States.

**Table 1:** Patient characteristics

Pt	Sex	Onset (y)^g^	Diagnosis (y)^g^	Examination (y)^g^	Wheelchair (y)	Ventilator (y)	FVC z-score sitting (% pred)	FVC z-score supine (% pred)	MRC %	CK U/l	GAA activity (nmol/h/mg)^h^	Allele 1	Allele 2
1^a^	M	0.5	2.5	10.04	Yes (11)	No	–0.25 (97 %)	–1.12 (87 %)	65 %	530	9.1	c.-32–13T > G (pm)	c.525delT (vs)
2^e, f^	F	0.8	0	0.1	No	No	Too young	Too young	n.a.	358	Deficient	c.-32–13T > G (pm)	c.2135 T > C (ls)
3	F	0.8	1.1	8.9	No	No	0.21 (102 %)	0.1 (101 %)	89 %	1871	13.3	c.-32–13T > G (pm)	c.923A > C (pls)
4^e^	M	0.8	2	2.4	No	No	Too young	Too young	n.a.	1353	13	c.-32–13T > G (pm)	c.2135 T > C (ls)
5	M	0.8	2.3	9.5	No	No	–1.53 (82 %)	–1.71 (80 %)	98 %	550	15.4	c.-32–13T > G (pm)	c.525delT (vs)
6	M	1	2	8.2	Partially (4)	No	–3.47 (59 %)	–4.36 (48 %)	79 %	3078	4.5	c.-32–13T > G (pm)	c.1051delG (vs)
7	M	1	2	13.7	No	At night (12)	–4.08 (54 %)	–5.4 (39 %)	87 %	548	17.9	c.-32–13T > G (pm)	c.525delT (vs)
8	M	1.5	2	13.3	No	No	–0.91 (90 %)	–1.76 (80 %)	93 %	1595	16	c.-32–13T > G (pm)	c.2481 + 102_2646 + 31del (vs)
9^a, f^	M	2	1	6.6	No	No	–0.97 (89 %)	–1.4 (84 %)	94 %	763	11	c.-32–13T > G (pm)	c.525delT (vs)
10	M	2.5	3	13	No	No	–3.01 (66 %)	–4.09 (54 %)	84 %	1960	8.6	c.-32–13T > G (pm)	c.2331 + 2 T > A (vs)
11^d^	M	5	10.8	10.8	No	No	0.2 (102 %)	–0.29 (97 %)	97 %	588	11.9	c.-32–13T > G (pm)	c.525delT (vs)
12	F	5	7.8	7.8	No	No	0.62 (108 %)	0.35 (104 %)	100 %	1003	11.6	c.-32–13T > G (pm)	c.2331 + 2 T > A (vs)
13^f^	M	5	2	7.6	No	At night (5)	–1.77 (78 %)	–1.72 (79 %)	100 %	436	Deficient	c.-32–13T > G (pm)	c.1062C > G (pls)
14	M	7	10	10.7	Yes (22)	At night (16)	–2.22 (75 %)	Unable	80 %	540	8,9	c.-32–13T > G (pm)	c.1548G > A (pls)
15^f^	M	8	4	15.8	No	No	–2.65 (70 %)	–3.05 (65 %)	93 %	1424	7.8	c.-32–13T > G (pm)	c.1441 T > C (pls)
16^f^	M	12	8	14.6	No	No	–2.43 (71 %)	Unable	82 %	1808	Deficient	c.-32–13T > G (pm)	c.307 T > G + c.271G > A (pls)
17	M	13	14	14.3	No	No	–1.68 (81 %)	–1.77 (80 %)	95 %	2935	6.2	c.-32–13T > G (pm)	c.1933G > A (pls)
18^f^	M	no symptoms	4	5.2	No	No	–1.41 (82 %)	–1.96 (76 %)	97 %	677	Deficient	c.-32–13T > G (pm)	c.2481 + 102_2646 + 31del (vs)
19^d, f^	M	no symptoms	13.1	13.1	No	No	–0.69 (92 %)	–1.77 (80 %)	100 %	614	Deficient	c.-32–13T > G (pm)	c.525delT (vs)
20^c, f^	M	no symptoms	14	15.2	No	No	3 (135 %)	1.72 (120 %)	n.a.	1409	Deficient	c.-32–13T > G (pm)	c.307 T > G (pls)
21^c, f^	M	no symptoms	16	17.1	No	No	1.29 (115 %)	–0.25 (97 %)	n.a.	1506	Deficient	c.-32–13T > G (pm)	c.307 T > G (pls)
22	M	0.5	1	1.3	Yes (4)	Died (10)^i^	–4.77 (45 %)^j^	Unable	n.a.	586	Deficient	c.1798C > T (ls)	c.525delT (vs)
23	F	1	1.9	12.5	Yes (6)	Yes (6)	Ventilator	Ventilator	10 %	1381	Deficient	c.875A > G (pm)	unknown/r.0?
24	M	2	2.9	2.9	Yes (6)	Died (6)^i^	–5.29 (33 %)^j^	Unable	n.a.	1046	Deficient	unknown	c.1645G > A (pm)
25	M	2.7	3.5	5.9	No	No	0.34 (104 %)	0.04 (100 %)	87 %	908	2.8	c.1634C > T (ls)	c.2481 + 102_2646 + 31del (vs)
26^b^	F	4	4	8.1	No	No	0.01 (100 %)	–0.63 (92 %)	98 %	572	2.3	c.-32–3C > G (ls)	c.1551 + 1G > A (vs)
27^b^	M	5	5	10.1	No	No	–1.38 (84 %)	–2.55 (71 %)	n.a.	774	1.7	c.-32–3C > G (ls)	c.1551 + 1G > A (vs)
28	F	6	7	9.9	Partially (9)	At night (8)	–6.36 (30 %)	–6.88 (25 %)	82 %	979	0.3	c.1829C > T (ls)	c.1912G > T (pls)
29	F	6.5	11.6	12.7	No	No	–2.36 (73 %)	–2.89 (67 %)	79 %	776	8.4	unknown (r.spl 2 %)	c.525delT (vs)
30	F	10	11	16.4	Yes (16)	Yes (12)	–8.07 (13 %)	Unable	72 %	1560	3.4	c.-32–3C > A (ls)	c.877G > S + c.271G > A (pls)
31^f^	F	no symptoms	15	15.9	No	No	1.33 (116 %)	1.2 (115 %)	100 %	1040	2.5	c.861C > T (r.spl = <5 %)	c.925G > A (pls)

Patients are listed by age of onset and are subdivided into two groups: those who carry the c.-32–13T > G mutation and those who do not ^a, b, c, d, e^ : Siblings; ^f^ patients who were diagnosed pre-symptomatically; ^g^: Age at onset, age at diagnosis, age at examination expressed in years (y); ^h^: GAA activity was deficient in all patients. Only results obtained in cultured fibroblasts and performed with the same method at Erasmus MC are reported; ^i^: both patients died of respiratory failure, one at age 6, the other at age 10; ^j^: first available lung function measurement (patient still untreated) at the ages of respectively 9 and 5.7 years; severity of the mutation is indicated by (vs) very severe; (pls) potentially less severe; (ls) less severe; (pm) potentially mild (for more information, see www.pompecenter.nl)

The median age at which patients had experienced their first symptom was 2.6 years (range 0.5–13y). At time of diagnosis, their median age was 4.0 years (range 0–16y). The commonest presenting symptoms were delayed motor development (in nine patients), and other symptoms related to limb-girdle weakness, such as frequent falling, difficulty climbing stairs, and problems with running and sports. Fatigue, persistent diarrhea and problems in raising the head in supine position were other first complaints. Median time span between symptom onset and diagnosis was 0.9 years (range 0 to 5.8 years).

Ten patients had been diagnosed pre-symptomatically. In six of them the diagnosis was made after elevated CK and transaminase serum levels had been found during a hospital admission for unrelated matters. The other four patients had been diagnosed because they had a sibling with Pompe disease. Five of these ten patients developed symptoms between diagnosis and first examination in our hospital (see for details Table 1).

### Clinical findings

All 31 patients were evaluated in the Pompe Center at Erasmus MC University Medical Center. Their ages at the time of examination ranged from 0.1 to 17.1 years. Table 2 shows the findings on clinical examination. Over 50 % of the 31 patients had low or absent reflexes, a myopathic face, and scoliosis. Facial muscle weakness was generally mild and did not lead to speech difficulties or dysphagia. One exception was a patient (patient 23 in Table 1) that had severe dysarthria and was fed via a percutaneous endoscopic gastrostomy catheter. This patient had been wheelchair bound and ventilator dependent since the age of 6 years.

**Table 2:** Results of clinical and neurological examination

	Number of patients (Total 31)
Clinical findings	
Low/absent reflexes	22 (71 %)
Weakness facial muscles	16 (52 %)
Scoliosis	16 (52 %)
Muscle tone decreased	13 (42 %)
Scapular winging	12 (39 %)
Muscle atrophy	11 (35 %)
Contractures	9 (29 %)
Low body weight	9 (29 %)
Ptosis	0 (0 %)
Physical limitations	
Standing up from supine position	18 (58 %)
Flexing the neck in supine position	17 (55 %)
Standing up from sitting on heels	13 (42 %)
Climbing stairs	13 (42 %)
Rising from a chair	10 (32 %)
Erecting back in prone position	8 (26 %)

Four deep tendon reflexes were tested: the biceps reflex, the triceps reflex, the knee-jerk reflex, and the ankle-jerk reflex

Nine patients had flexion contractures, mainly in the ankles. Three of the nine patients had contractures of the hips and knees. Several patients had undergone corrective surgery for either contractures (*n* = 4, patients 1, 6, 10, 29 in Table 1), or scoliosis (*n* = 4, patients 7, 14, 23, 30 in Table 1). It is noteworthy that 29 % of the patients were underweight; corrected for height, their weight was 4.3 to 2.0 standard deviations below healthy peers.

Standardized neurological examination of all patients showed that 70 % had one or more physical limitations. Over 50 % of all patients had difficulties standing up from supine position and flexing the neck in supine position (Table 2). Other important limitations were problems with climbing stairs, rising from a chair and standing up from sitting on their heels.

### Distribution of muscle weakness

**Fig. 1:**
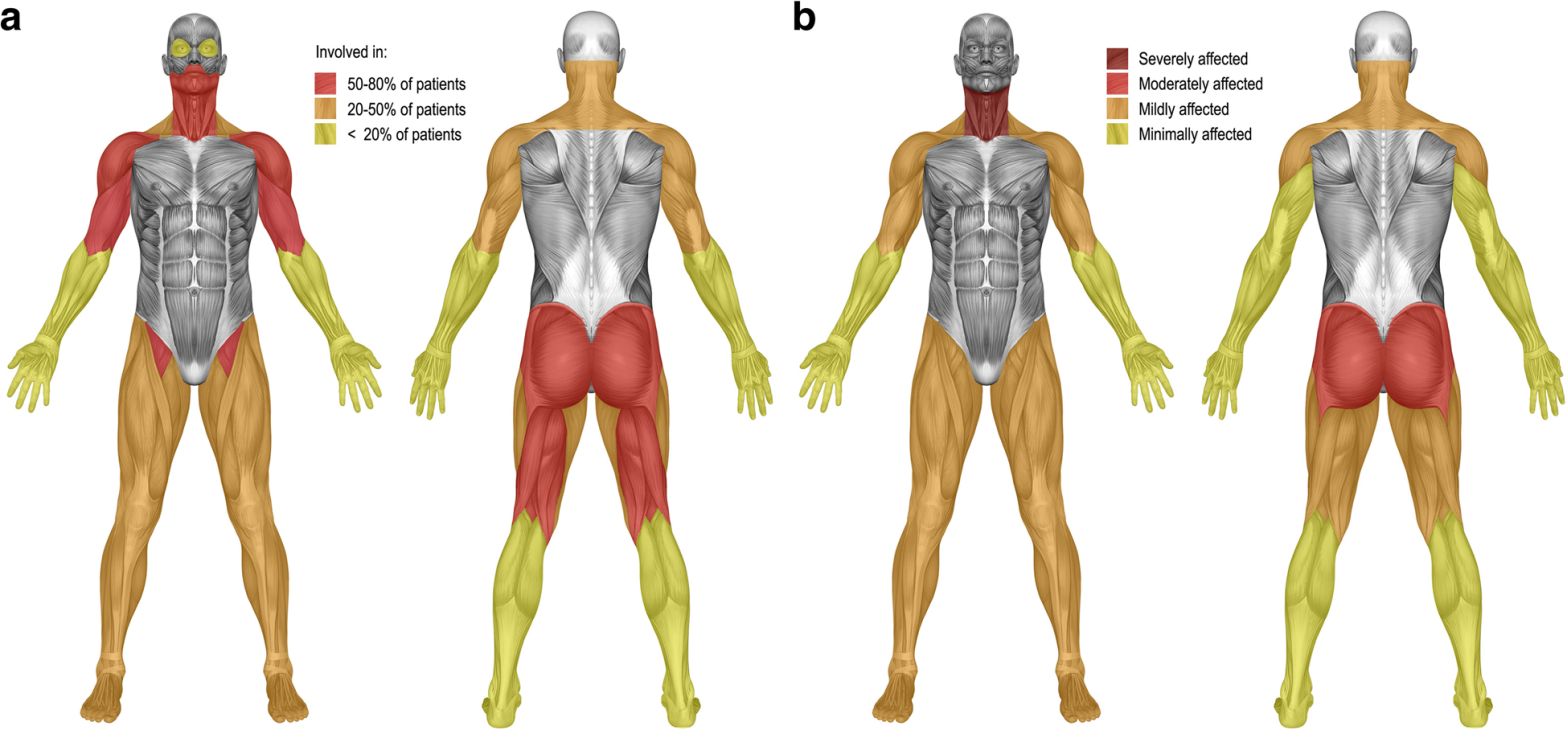
Distribution of skeletal muscle weakness (**a**) and severity of weakness of the individual muscles (**b**)

Figure 1 shows the severity of muscle weakness and in how many patients the various muscle groups were affected. The commonest weakness was in the neck flexors, which were affected in 75 % of the patients. Other muscles that were frequently affected were the gluteus maximus (extension of the hip), the ileopsoas (flexion of the hip), the biceps and the deltoid muscle. The triceps, wrist extensors, and foot plantar flexors were relatively unaffected. In patients with far advanced disease, all muscles were affected.

All patients had a lower total HHD sum score than age related peers. Total muscle strength ranged from 0–79 % of normal (median 55 %). Four patients were wheelchair-bound at the time of investigation; four others became wheelchair dependent in the period thereafter. The ages of these eight patients when they became wheelchair dependent ranged from 4 to 22 years (*n* = 8, median 7.5 years). The median time period between first symptoms and loss of ambulation was 4.5 years (*n* = 8, range 3 to 15 years).

### Lung function testing

**Fig. 2:**
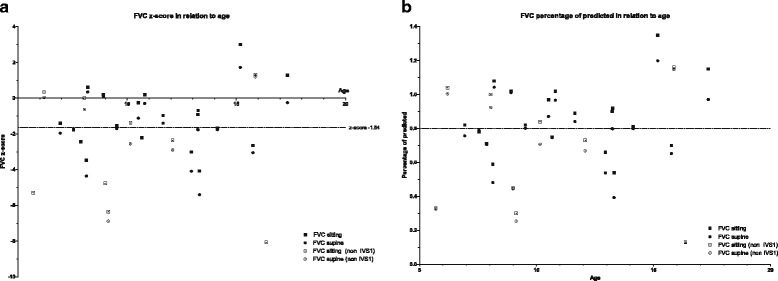
FVC z-score (**a**) and percentage of predicted (**b**) in sitting and supine position

In all subjects, FVC values were equal or higher than slow VC. Hence, all analyses were done on FVC. In sitting position, 14 of the 29 patients (48 %) had decreased forced vital capacity (FVC) indicated by z-score below −1.64 (13 patients had a percentage of predicted below 80 %). In one of these patient the FVC was too low to be measured reliably in sitting position. In supine position, 19 of the 29 (66 %) had a FVC z-score below −1.64 (18 patients had a percentage of predicted below 80 %). In 6 of these patients, lung volume was too low to be measured reliably in supine position. Lung function measurements are displayed in z-scores and percentage of predicted in Fig. 2. FEV1/VC ratios were normal in all patients, indicative of a restrictive abnormality.

The median difference in FVC between sitting and supine positions (postural drop) was 6.0 % (range 0 to −18 %). Five patients were ventilator dependent at the time of first evaluation, and one became ventilator dependent during follow-up (see Table 1 for details). Five of these six patients had a scoliosis. At the ages of 6 and 10 years, two other patients died from respiratory failure, when it was decided not to start respiratory support. The median duration from first symptoms to any kind of respiratory support or death by respiratory insufficiency was 4.5 years (range 0 to 11 years, *n* = 8). The median age at start of ventilation or death was 9 years (range 5 to 16 years, *n* = 8).

### Cardiac evaluation

Cardiac evaluation showed abnormalities in six patients; in three, the findings were considered to be related to Pompe disease. Two patients had hypertrophic cardiomyopathy without outflow-tract obstruction (patients 22, 23 in Table 1). In one patient, this had first been noticed at one year of age, and in the other at two. Their ECGs showed high amplitude QRS complexes and repolarization disturbances consistent with their hypertrophic cardiomyopathy. In addition, their ECGs and that of a third patient (patient 3 in Table 1) showed a short PR interval and a delta wave suggestive of Wolff-Parkinson-White syndrome.

Minor abnormalities of the cardiac valves were noted in three patients. The abnormalities included a quadricuspid aortic valve, a minor deformity of the tricuspid valve leading to minimal tricuspid regurgitation, and minimal insufficiency of both atrioventricular valves. These abnormalities were all considered to be coincidental findings and not related to Pompe disease.

### Enzymatic and molecular diagnosis

Table 1 shows the patients’ genotypes and the severity of their mutations. All mutations have been described previously in the literature (www.pompecenter.nl). Twenty-three patients (74 %) carried a potentially mild mutation on one *GAA* allele and a severe mutation on the other. Twenty-one of these patients carried the common c.-32–13T > G splice-site mutation. Since none of the mutations in the other eight patients were considered potentially mild, these patients’ genotypes were considered to be more severe. Acid α-glucosidase activity measured in cultured fibroblasts ranged from 0.3 to 17.9 nmol/h/mg protein (*n* = 21, median = 8.6; control range 45–160 nmol/h/mg). In patients with the c.-32–13T > G/‘null’ genotype activity ranged from 4.5 to 17.9 nmol/h/mg (*n* = 14, median = 11.3) and from 0.3 to 8.4 nmol/h/mg (*n* = 7, median = 2.5) in patients with the non-IVS1 genotype.

### Laboratory parameters

At first visit, all patients had elevated CK levels (median 979 U/l, range 358 to 3078 U/l; normal values below 230 U/l)). Transaminase levels were elevated in all 31 patients as well, including those who were symptom-free; AST ranged from 82 to 610 U/l (normal values below 51 U/l), ALT from 71–551 U/l (normal values below 39 U/l). LDH levels were elevated in 21 out of 30 patients, ranging from 449 to 2828 U/l (normal values below 765 U/l).

### Comparison of patients with the c.-32–13T > G/‘null’ genotype (IVS1) and those with other genotypes (non-IVS1)

Comparison of the twenty-one patients with the c.-32–13T > G/‘null’ genotype and the 10 patients with other mutations showed no significant differences in age at first symptoms and age at diagnosis (Table 3). Their median age at first examination was similar. We noted that patients in the non-IVS1 group tended to have lower muscle strength and a more severely restricted lung function in sitting and supine positions than those in the IVS1 group. This was in line with the fact that more non-IVS1 than IVS1 patients had become wheelchair bound and ventilator dependent at a relatively young age. In addition, the two patients with hypertrophic cardiomyopathy belonged to the non-IVS1 group. Another interesting observation was that 18 out of the 21 patients (86 %) with the c.-32–13T > G/‘null’ genotype were male, against 4 out of 10 in the non-IVS1 group. Other than the possible trend that patients with the c.-32–13T > G/‘null’ genotype were generally less severely affected compared to patients with other genotypes we weren’t able to identify other genotype - phenotype relations in the c.-32–13T > G/‘null’ genotype group.

**Table 3:** Comparison of patients with the c.-32–13T > G mutation and other mutations at the time of examination

	All	c.-32–13T > G	Other mutations	*P*-value
Patients	31	21	10	–
M/F	22/9	18/3	4/6	
Age first symptom (median)	2.6 (0.5–13)	2 (0.5–13)	4.0 (0.5–10)	0.6
Age diagnosis (median)	4.0 (0–16)	3.0 (0–16)	4.5 (1–15)	0.6
Age at examination (median)	10.1 (0.1–17.1)	10.7 (0.1–17.1)	10 (1.3–16.4)	0.7
Disease duration (median)	5.1 (0.8–12.7)	6.5 (1.3–12.7)	4.1 (0.8–11.5)	0.2
Diagnosed pre-symptom.	10	9	1	–
Still symptom free	5	4	1	–
HHD sumscore (%)	55 (*n* = 24)	58 (*n* = 18)	36 (*n* = 6)	0.02
FVC pred sitting (%)^a^	82	82	58	0.15
FVC pred supine (%)^a^	79	80	55	0.08

Disease duration is calculated as time between the presentation of first symptoms and first examination in our hospital

^a^For patients who didn’t perform lung function testing in supine position the value obtained in sitting position was used. For one patient using 24 h invasive ventilation a value of 0 % was used in the analyses

## Discussion

As little information is available on the clinical presentation of children with non-classic forms of Pompe disease, we evaluated 31 children’s clinical and molecular characteristics. Our findings highlight that non-classic Pompe disease can cause a significant burden in childhood and add to the understanding that Pompe disease presents as a broad spectrum of clinical phenotypes.

The presentation of Pompe disease can be variable. Children in our patient population typically presented with weakness of the limb-girdle muscles and/or delayed motor development; lung function was compromised in approximately half of them, and 2 patients whose muscle function deteriorated very rapid also had hypertrophic cardiomyopathy.

Nevertheless, the diagnosis of Pompe disease should also be considered in children whose symptoms are less typical, such as disproportional weakness of the neck flexors, unexplained fatigue, persistent diarrhea, and an elevation of transaminase levels. Independent of whether the children had symptoms or not, all patients participating in the study had elevated CK, ALT and AST values. It should be noted, however, that in rare cases CK, ALT and AST may be normal, as we very recently encountered in a childhood onset patient (personal communication) and as was earlier described for about 10 % of adult patients [28].

Although respiratory problems did not precede proximal muscle weakness in any of the children, lung function was already significantly restricted in 48 % of the cases. In 26 % of the affected children, respiratory insufficiency either led to the need for ventilator support or resulted in death during childhood. This finding signifies the importance of early monitoring of lung volume by means of spirometry in children affected by Pompe disease as also advised in adults [17, 29].

Lung function tests are best performed in both sitting and supine positions, as 9 patients had postural drops suggestive of diaphragm weakness. This is a well-known feature of Pompe disease, and contributes to the onset of nocturnal hypoventilation [29, 30]. Since a recent study in children with neuromuscular disorders found that daytime lung function and nocturnal hypoxemia were poorly correlated, we suggest to regularly perform sleep studies as an additional tool for identifying children with nocturnal hypoventilation [31].

Twenty-two of the 31 children that we investigated were male. Interestingly, among them were 18 with the c.-32–13T > G/‘null’ genotype that is most common among adult patients (against only 3 of the 9 female patients). In two studies that focused on disease variation among children and adults with the c.-32–13T > G/‘null’ genotype, the male-to-female distribution was equal (55 and 58 % males, respectively). However, neither study focused on any potential difference in age at onset between male and female patients [12, 13]. The present study indicates the existence of such a gender difference. Our earlier analysis of 225 published case reports on children and adults with Pompe disease also showed a predominance of males (67 %) in patients under 18 years old [11]. Previous studies reported that pulmonary function was more affected in males than in females [17], and that more men than women had bulbar involvement and shoulder-girdle muscle weakness [17]. A study comparing phenotypes in siblings with Pompe disease also confirmed that males were more severely affected than females [32].

Since Pompe disease is inherited as an autosomal recessive trait, there has been no satisfactory explanation to date why males with the same *GAA* genotype as females would present at an earlier age. This finding seems to suggest that the clinical expression of Pompe disease involves secondary gender-related factors. Gender differences have also been reported for other neuromuscular disorders such as facioscapulohumeral muscular dystrophy and some subtypes of limb-girdle muscular dystrophy [33–35]. One muscle related difference between men and women found so far is that women with limb girdle muscular dystrophy type 2A and 2B showed less muscle fiber atrophy compared to males [35]. This may also apply in Pompe disease. Though other causes have been suggested, such as differences in genetic and epigenetic factors, the exact mechanism remains elusive.

Our findings are fully consistent with the broad spectrum of clinical phenotypes associated with the c.-32–13T > G/‘null’ *GAA* genotype [12, 13]. Looking at the genotype-phenotype correlation within our group of children shows a similar age of onset, age at diagnosis, and current age for all different genotypes. Several patients were wheelchair bound and/or ventilator dependent despite of having the c.-32–13T > G/‘null’ genotype, which is mostly associated with adult onset disease. The c.-32–13T > G, is a leaky splice site mutation that results in the formation of 10–20 % of normally processed alpha-glucosidase protein and activity, which explains the later onset non-classic phenotype in patients c.-32–13T > G/‘null’ genotype. In our group of patients the second mutation (severe, less severe or potentially less severe) did not seem to have an effect on the age of presentation. This is in line with earlier studies of Kroos et. al., Wens et.al. and Montalvo et. al. also showing a broad variation of phenotypes among patients with the c.-32–13T > G/null genotype. It was hypothesized that epigenetic factors and environmental factors influence the level of disease severity [12, 13, 32]. More research is required.

At a group level, children in the non-IVS1 group seem to be more severely affected than patients with the 32-13T > G/‘null’ genotype. This is illustrated by two specific patients in the non-IVS1 group who had hypertrophic cardiomyopathy and became fully wheelchair bound; one at the age of 4 years and the other at the age of 6. The first child died from respiratory failure at the age of 10. The other child became completely ventilator dependent when she was 6 years old. These two patients expressed a phenotype that Slonim et al. previously called the “atypical infantile form” of Pompe disease [18].

Overall, the genotypes identified in the non-IVS1 group were more severe than those in the IVS1 group (see Table 1 and www.pompecenter.nl). Some of the genotypes have been described in the literature, such as the genotype c.1634C > T/c.2481 + 102_2646 + 31del (our patient 25). The c.1634C > T (ls) in combination with a very severe mutation was previously described in three patients. One of those patients presented at the age of one year, became dependent on respiratory support at the age of 20, and was wheelchair bound at the age of 23 [15]. The second patient was diagnosed at 16 years of age, and began to use a walking stick at 19. Pulmonary function worsened at the age of 17–19 years and vital capacity dropped to 26 % of predicted and became respirator dependent at the age of 20 years [36]. The third patient presented at the age of 13 years with pronounced limb girdle weakness and died at the age of 18 years [37]. The similarities in clinical course of these previously reported cases are remarkable, and if the genotype-phenotype correlation holds for our patient, who was only 6 years old at time of examination, he carries a high probability to develop severe respiratory and mobility problems before adulthood. Such information may be relevant when it is time to decide when to start enzyme replacement therapy.

Children and adults share a wide variation of disease presentation and disease progression, and a similar involvement of respiratory and proximal skeletal muscles [8, 9, 12–16]. Although the distribution of muscle weakness shows a limb-girdle pattern in both children and adults, there are also differences. While the neck flexors are by far the most severely affected muscle group in children, they are only mildly affected in adults [8, 15]. A recent MRI study performed in 20 adult patients by Carlier et al. also showed relatively mild involvement of the neck flexors in adults [38].

Another difference is the relative sparing of the quadriceps muscle in adult patients [28, 38]. In the current population of 31 children with Pompe disease, the muscles of the thigh were affected more heterogeneously, and the quadriceps muscles were not spared. Neither did any of our patients have ptosis, despite a recent publication in which ptosis was present in 14.7 to 23 % of adult Pompe patients [28, 39, 40]. It should be noted that ptosis has often been found in an early stage of the disease, even as a presenting symptom in adult Pompe patients. While van der Beek et al. found that patients had difficulties with speech, chewing or swallowing, which was suggestive of bulbar weakness in 28 % of their patients, we found bulbar weakness in only one patient. In contrast, 52 % of our children had scoliosis, compared to only 21 to 23 % of adult Pompe patients [11, 28]. In several children the scoliosis was so severe that it interfered with their mobility and lung volume; four children needed surgical correction of the spine. A cross-sectional analysis of data from the Pompe Registry, a large multinational observational program, found scoliosis to be present in 57 % of patients with childhood disease onset [41].

Our study had two main limitations. First, since the Pompe Center at Erasmus MC University Medical Center serves as a national and international referral center for Pompe disease, there may be selection bias, due to referral of patients who were more than average severely affected. Nonetheless, ten of the 31 patients had been diagnosed pre-symptomatically, 5 of whom were still symptom-free at time of evaluation. A second limitation is the fact that the study was cross-sectional. As all children manifesting significant symptoms of the disease, started to receive enzyme replacement therapy during follow-up, the approval of this therapy in 2006 interfered with the collection of longitudinal follow-up data.

## Conclusions

In conclusion, our study shows that the course of childhood Pompe disease varies widely, and patients may manifest serious problems before adulthood. We stress that Pompe disease should be considered in the differential diagnosis of patients with less familiar signs such as disproportional weakness of the neck flexors, unexplained fatigue and persistent diarrhea. Disease presentation, distribution of muscle weakness, and the occurrence of specific symptoms such as bulbar muscle weakness or ptosis all appear to be different from those in adult patients. Regular assessment of lung volume and sleep studies are recommended to identify children at risk for early respiratory insufficiency. Patients with mutations other than the c.-32–13T > G were overall more severely affected, which is consistent with their more severe genotypes. The majority of affected children with *GAA* genotype c.-32–13T > G/‘null’ appeared to be male.

### Ethics approval and consent to participate

All procedures followed were in accordance with the ethical standards of the responsible committee on human experimentation (institutional and national) and with the Helsinki Declaration of 1975, as revised in 2000. Informed consent for inclusion in the study was obtained from all patients and/or their parents.

### Consent for publication

Not applicable

### Availability of data and material

All available data is published in the manuscript. Individual patient records can’t be made available due to patients’ privacy.

## Abbreviations

ALT: alanine aminotransferase
AST: aspartate aminotransferase
ATS: American thoracic society
CK: creatinine kinase
ERS: European respiratory society
FEV1: forced expiratory volume
FVC: forced vital capacity
*GAA*: acid a-glucosidase gene
HHD: hand held dynamometry
LDH: lactate dehydrogenase
MMT: manual muscle testing
VC: vital capacity
